# Cases in a series of carcinoid syndrome and carcinoid heart disease

**DOI:** 10.5830/CVJA-2018-040

**Published:** 2018

**Authors:** R Matshela Mamotabo

**Affiliations:** University of KwaZulu-Natal, Durban, South Africa; Mediclinic Heart Hospital, Pretoria, South Africa; London School of Economics and Political Science, London, UK

**Keywords:** carcinoid syndrome, carcinoid heart disease, 5-hydroxyindoleacetic acid

## Abstract

Although carcinoid syndrome is regarded as a rare entity, carcinoid patients with evidence of cardiac involvement show a markedly reduced survival time. Patients with advanced signs of right-sided heart failure represent a subgroup at particularly high risk. Echocardiography remains the gold standard to diagnose or confirm structural cardiac involvement in patients with underlying carcinoid disease. This is the notion that propelled us to report on cases of carcinoid syndrome with cardiac involvement. We also review carcinoid syndrome and carcinoid heart disease, and challenges regarding the diagnosis and management of carcinoid heart disease.

Carcinoid heart disease (CHD) has previously been reported as a rare form of valvular heart disease, mostly associated with metastatic carcinoid tumour. Most systemic manifestations of carcinoid tumours are related to the release of vasoactive substances from the tumour, including serotonin and other circulating humoral substances. Although CHD is presumed to be rare, we use this opportunity to report on a collection of interesting patients with CHD, who presented differently and posed challenges in their short- and long-term management.

## Case report

Seven patients with carcinoid and cardiac involvement are summarised in Table 1. However, two of these patients with classical echocardiographic images were extensively reviewed and are reported on below.

**Table 1:** Summary of patients with confirmed carcinoid syndrome and carcinoid heart disease

*Parameters*	*Patient 1*	*Patient 2*	*Patient 3*	*Patients 4*	*Patient 5*	*Patient 6*	*Patient 7*
Age, years	78	32	81	55	63	71	68
Gender	Male	Male	Female	Female	Male	Male	Female
Urinary 5-HIAA, μmol/24 h	406–548	465–269	< 300	< 200	< 200	< 200	< 200
Echo features	Restrictive TV leaflet motion, torrential TR	Restrictive TV and PV leaflets motion	Moderately restrictive TV leaflet motion	Mildly restrictive TV leaflet motion	Moderately restrictive TV leaflet motion	Mildly restrictive TV leaflet motion	Mildly restrictive TV leaflet motion
Octreotide scan	+	+	+	+	+	+	+
Hepatic lesions on CT	Multiple	Multiple	One	None	Multiple	None	None
Management	Declined surgery	TVR	Medical Management	Medical Management	Medical Management	Medical Management	Medical management
Follow up	Died	Improved	Died	Improved	Improved	Improved	Improved

5-HIAA, 5-hydroxyindole acetic acid; TV, tricuspid valve; TR: tricuspid regurgitation, TVR, tricuspid valve replacement; PV, pulmonary valve.

Patient 1: The first patient was a 78-year-old African male who first presented to his local hospital with constitutional symptoms and abdominal distension, which progressed over a four-month period. He was later referred to us for further management. This was his first-ever consultation and admission to any medical facility. There was no past surgical or medical history of note and no history of illicit drug use. However he was an occasional drinker and a smoker with a three-pack year history. He had no family history of note.

**Fig. 1:**
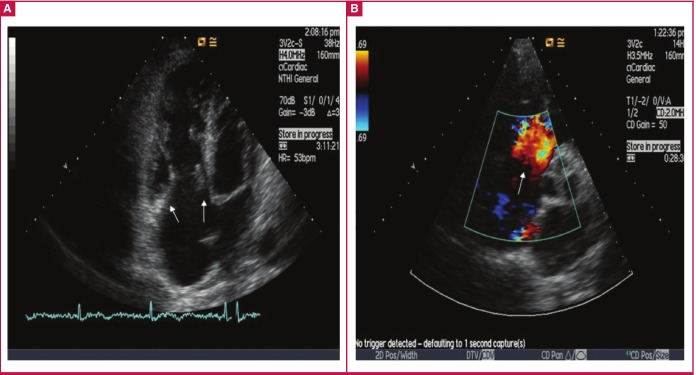
Patient 1. (A) Apical four-chamber view showing tricuspid leaflets that are thickened and retracted (arrows). The right ventricle and atrium are dilated. (B) Colour Doppler with free flow through the tricuspid valve during systole in a parasternal short-axis view at the level of the aortic valve (arrow).

Physical examination revealed skin hyperpigmentation, lower abdominal mass and features of severe tricuspid regurgitation with right heart failure. The rest of his clinical examination was unremarkable. Carcinoid syndrome with CHD was suspected during routine transthoracic echocardiographic assessment, and his images are shown in Fig. 1.

His biochemical laboratory results revealed a markedly elevated serum 5-hydroxyindoleacetic acid (HIAA), which was more than 10 times the upper limit of normal. An octreotide scan was positive for the primary lesion localised around the periprostatic area. Additional blood results revealed normal renal and hepatic function. His full blood count revealed features in keeping with anaemia of chronic diseases. The prostate-specific antigen level was mildly elevated. Chest radiography revealed a mildly increased cardiothoracic ratio and hyperinflated lungs. Unfortunately, the patient refused further hospital management, including surgery, and died a year later.

Patient 2: The second patient was a 32-year-old male of Indian descent who presented with a two-month history of abdominal pain, weight loss and diarrhoea. He had no significant past medical, surgical, family or occupational history. Patient 2: The second patient was a 32-year-old male of Indian descent who presented with a two-month history of abdominal pain, weight loss and diarrhoea. He had no significant past medical, surgical, family or occupational history.

His clinical examination revealed features of right heart failure with severe tricuspid and moderate pulmonary valve regurgitation. The rest of his examination was unremarkable. Chest radiography revealed a mildly increased cardiothoracic ratio and an electrocardiogram revealed sinus rhythm. Blood results revealed mild pre-renal dysfunction, anaemia of chronic disease, normal liver function test and normal comprehensive metabolic panel. Further biochemical results revealed an elevated 5-HIAA level and prior to referral, his private practitioner had already commenced medical therapy, which included octreotide.

**Fig. 2:**
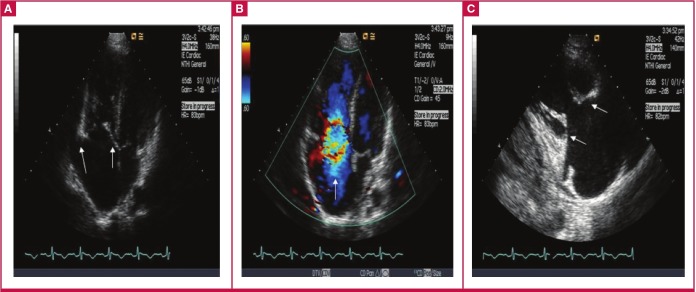
Patient 2. (A) Apical four-chamber view: note the thickened, immobile and retracted tricuspid leaflets and minimally thickened mitral valve leaflets (arrows), and the dilated right atrium and ventricle. (B) Torrential tricuspid regurgitation (note the arrow). (C) Marked failure of coaptation (indicated by arrows) of the tricuspid valve leaflets.

**Fig. 3:**
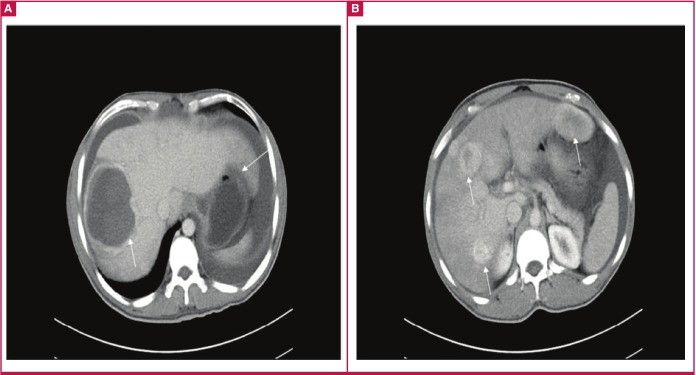
Patient 2. Computed tomography scan. Notice a large echogenic mass on the right lobe of the liver, and further multiple echogenic masses on both lobes (arrows).

Two weeks later the patient was referred for a specialist’s opinion and further management. His echocardiographic and computed tomographic images are presented in Figs 2 and 3, respectively. His symptoms improved dramatically on medical therapy and he was subsequently referred to the surgical team, where an elective tricuspid valve replacement (TVR) was successfully performed six months later. His intra-operative and postoperative periods were uneventful, and his symptoms continued to improve on subsequent follow-up visits.

## Discussion

**Prevalence of carcinoid syndrome and CHD**

Reports have indicated that at least 50% of patients with clinical manifestations of carcinoid syndrome present with echocardiographic evidence of cardiac or cardiovascular involvement.^1^-^5^ At least a quarter of carcinoid patients with cardiac manifestations present with right-sided cardiac disease. Although CHD is undoubtedly regarded as a rare entity, it is an interesting and important cause of intrinsic tricuspid and pulmonary valve disease and is associated with significantly high morbidity and mortality rates. Tricuspid and pulmonary valve regurgitations usually occur as secondary phenomena due to dilatation of the valve annular ring, secondary to right ventricular failure or as a result of severe pulmonary hypertension.

Previous reports have indicated that the incidence of carcinoid tumours occurs at a rate of 1.2 to 2.1 in 100 000 of the general population.^6^,^7^ In most instances, at the time of diagnosis, 20 to 30% of patients present with carcinoid syndrome and approximately 50% of these patients develop CHD, which typically causes abnormalities of the right side of the heart.^5^,^8^,^9^ In an estimated 20% of patients with carcinoid tumours, CHD is the primary presentation of the metastatic carcinoid disease.^5^,^8^,^9^

Although it is usually believed that carcinoid tumours that have hepatic involvement are highly associated with pathological cardiovascular damage, particularly right-sided cardiac involvement related to the large amount of metabolic products reaching the heart, a small proportion of patients, around five to 10%, present with significant left-sided disease due to direct blood flow from the right to the left side of the heart, or in some cases related to the presence of a primary lung tumour. In addition, cardiac manifestations of carcinoid syndrome could also be related to the paraneoplastic effects of vasoactive substances released by malignant cells rather than any direct metastatic involvement of the heart. Most importantly, patients with progressive cardiac disease tend to have higher levels of these vasoactive substances compared to those without cardiac disease.

**Cardiac and cardiovascular structural changes in CHD**

Typical pathological features of CHD are plaque-like deposits of fibrous tissue deposited on the endocardium of the valvular cusps and leaflets, atria and ventricles, sometimes involving the downstream aspects of the tricuspid and pulmonary valves, endocardium of the cardiac chambers, intima of the vanae cavae, pulmonary artery and coronary sinus (Figs 1, 2).

Although the fibrous tissue may result in distortion of the valves, the morphology of the valve leaflets is classically not disrupted. However, the endocardial thickening may lead to valve retraction and fixation (Figs 1, 2).

The tricuspid valve is most commonly involved, with typical tricuspid valvular regurgitation and rarely stenosis. The pulmonary valve is the second most commonly affected, presenting as mixed pulmonary valve disease. Pulmonary stenosis is more frequently noted, compared with tricuspid stenosis, due to the smaller orifice of the pulmonary valve and also due to plaque deposition on the pulmonary valve, within the pulmonary annulus and sinuses, results in narrowing of the pulmonary roof.

In rare situations, primary ovarian carcinoid disease may lead to CHD without evidence of hepatic metastases because the ovarian vein drainage bypasses the portal circulation. This pathognomic mechanism was previously reported in four patients from the Mayo Clinic.^10^ The sub-valvar apparatus, including the tendinous chords and papillary muscles of the mitral valve, could also be affected in rare instances where the left side of the heart is involved. The mitral and/or aortic valves are frequently involved in patients with right-to-left shunt or primary bronchial carcinoma. Metastatic involvement of the myocardium is uncommon; however, it has been reported.^11^

The Mayo Clinic previously reported 132 carcinoid syndrome patients, where a total of 74 (56%) patients had echocardiographic evidence suggestive of CHD. A total of 62 (90%) of the 74 patients had moderate to severe tricuspid valve regurgitation and 36 (49%) developed thickened, retracted, immobile pulmonary leaflets.^10^ In the same report, pulmonary regurgitation and stenosis were demonstrated in 81 and 53% of the patients, respectively.^10^ Only five patients (7%) had left-sided CHD and four (5%) of these had a patent foramen ovale or lung carcinoma.^10^ Myocardial metastases were also reported in only three (4%) patients and 10 (14%) had small pericardial effusions.

**Natural history and controversies of CHD**

Cardiac involvement in carcinoid syndrome heralds a decline in clinical outcome with poor survival rate without treatment. The previous three-year mortality rate data on CHD patients had indicated only a 31% survival rate, compared with approximately twice the survival rate for patients without cardiac involvement.^5^ Treatment of the cardiac aspects of carcinoid syndrome improves symptoms and increases longevity in carcinoid patients.^4^

Previously, a small study evaluated and reported on a total of 71 patients with carcinoid syndrome, who had serial echocardiograms performed a year apart, and retrospectively assessed factors that were associated with progression of cardiac disease.^8^,^9^ Although serotonin level was associated with progression of CHD, the risk of progressive heart disease was also higher in those patients who received chemotherapy than in those who did not, which is very controversial.^8^-^9^

In addition, Denney et al.^12^ reported that patients with carcinoid syndrome in whom heart disease developed had higher levels of serotonin before and after treatment with somatostatin analogue, compared with patients without cardiac lesions. Their data also reported similar finding in patients with pre-existing heart disease not related to carcinoid syndrome. In addition, peaked levels of 5-HIAA were also a significant predictor of progressive CHD and were reported to be markedly increased in patients with severe symptomatic heart disease who were referred for cardiac surgery.^8^,^12^-^16^

**Diagnosis of CHD**

Clinical presentations: usually a high index of clinical suspicion is needed to diagnose CHD. The time interval from the onset of carcinoid symptoms to the diagnosis of CHD is usually approximately two years, however it may take as long as five years.^5^ Patients with florid or classical carcinoid symptoms have a 50% chance of cardiac involvement.^5^

The physical examination will usually reveal features of regurgitant lesions and most commonly a pansystolic murmur of tricuspid regurgitation along the left sternal border. In some cases, there may be a concomitant murmur of pulmonary stenosis or regurgitation or both. A careful interpretation of the jugular venous pressure is crucial when assessing patients with suspected CHD. The classical large V wave may be the first finding on physical examination, suggestive of significant tricuspid regurgitation.

Biochemical screening: clinical suspicion of carcinoid syndrome or CHD usually leads to further evaluation, including biochemical screening, with the measurement of urinary 5-HIAA excretion. The biochemical measurement of 24-hour urinary 5-HIAA excretion has shown a sensitivity and specificity of 75 and 100%, respectively, for the diagnosis of carcinoid syndrome.^7^ Measurement of blood serotonin levels of an alternative biochemical marker such as plasma chromogranin A may be helpful if the urinary test is inconclusive.

Chest radiography: a chest radiograph and electrocardiogram have limited value in diagnosing CHD. A chest X-ray is usually normal in at least 50% of patients and in the remainder it may be non-specific. Other radiographic features in carcinoid patients with CHD include cardiac enlargement and pleural effusions or nodules.

Electrocardiography:^5^,^8^,^15^ in most cases, electrocardiograms are normal in patients with carcinoid syndrome or CHD, however, in severely symptomatic patients, usually low QRS voltages or poor R-wave progression have been reported, and this usually occurs in patients with advanced cardiac disease. The non-specific abnormal findings in CHD patients may include ST–T-wave abnormalities, sinus arrhythmias, P pulmonale or right bundle branch block.

Transthoracic echocardiography (2D-TTE): this remains the gold standard during the initial diagnostic evaluation of any patients suspected of CHD. Classical 2D-TTE findings will include thickened and shortened tricuspid valve (TV) leaflets. Similar to our two cases, the TV leaflets may become severely retracted, with reduced leaflet mobility. In most instances, the septal and anterior TV leaflets are predominantly affected (Figs 1, 2). In advanced stages of TV disease, the leaflets may become fixed in the semi-open position (Figs 1, 2).

**Fig. 4:**
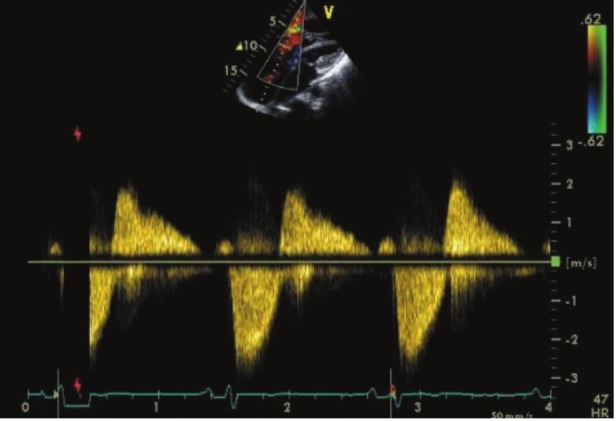
Continuous-wave Doppler demonstrating a daggershaped pattern in a patient with severe tricuspid regurgitation due to carcinoid heart disease, with early peak pressure and a rapid decline. In addition, the patient had marked failure of coaptation (FOC) resulting from severely thickened and retracted tricuspid valve leaflets due to underlying carcinoid disease (image courtesy of Fox and Khattar).^5^

Similar to the tricuspid valve, pulmonary valvular cusps usually appear thickened, with retraction and reduced leaflet mobility, and it may be difficult to visualise them during routine echocardiographic evaluation. Doppler echocardiographic assessment of the pulmonary valve may be helpful as there are challenges related to difficulties in demonstrating anatomical changes. The right atrium and ventricle may become increasingly dilated over time (Figs 1, 2). A dagger-shaped profile with an early peak velocity and a rapid decline, which indicates rapid pressure equalisation between the right atrium and ventricle, is a common finding during continuous-wave Doppler tracing in CHD patients presenting with severe tricuspid regurgitation (Fig. 4).

**Management strategies for CHD**

The principal management strategies in CHD patients should strictly focus on primary treatment of right heart failure, therapy to reduce secretion of tumour product, and valve surgery.

Medical management: medical treatment modalities for right heart failure in patients with CHD include standard heart-failure therapy. However these strategic approaches have not been proven effective in most patients with CHD.

Somatostatin analogue, particularly octreotide, has a direct effect on reducing the vasoactive peptides and has demonstrated direct clinical benefit and biochemical improvement. Alternatively, lanreotide, which has the advantage of less frequent administration compared with octreotide, may be a good option. Reports have indicated that somatostatin analogue has demonstrated symptomatic relief and a decrease in measurable urinary 5-HIAA excretion and serum serotonin concentration.^10^

Newer treatment options include the leukocyte interferonalpha, which may be used in conjunction with somatostatin antagonist. However data on this treatment option are limited.

Cardiac surgery: asymptomatic patients or those who exhibit minimal symptoms usually need closer follow up with regular transthoracic echocardiography and exercise testing to assess their functional status. Patients who develop severe cardiovascular symptoms related to CHD should be evaluated for valve-replacement surgery. For suitable candidates, valve surgery is the only definitive curative treatment modality for severe heart failure. Although reports have implicated balloon valvoplasty as an alternative and it has produced symptomatic improvement in some patients with stenotic valve lesions related to CHD, data on its application in CHD patients are limited.^17^

Indications for surgery: right heart-failure symptoms, severely impaired exercise capacity, progressive right ventricular enlargement or decline in right ventricular systolic function are some of the indications for surgical intervention in CHD patients. However, some patients with severe CHD may require cardiac valve surgery despite minimal cardiac symptoms.

Although tricuspid mechanical valve prostheses may be considered adequately durable and relatively unaffected by vasoactive substances, bioprosthetic valves may be preferable since anticoagulants can be avoided.^17^-^39^ Due to high bleeding tendencies in patients with hepatic metastases, bioprosthetic valves would be the best option for this group of patients. However, the life expectancy of bioprosthetic tricuspid valves is likely to be shorter than that of mechanical valves, particularly in CHD. Tricuspid valve repair could be an option and important area of future research, however currently, tricuspid valve repair does not seem feasible in CHD.

Pulmonary valvectomy or valve replacement is preferred for those with pulmonary valve disease secondary to carcinoid disease. Although pulmonary valve replacement may reduce the risk of right heart dilatation postoperatively, larger studies with more convincing results are warranted.

Successful surgical intervention has been associated with an improvement in survival rate and quality of life in those patients who were successfully treated surgically. Despite this premise, older patients (over the age of 60 years) remain a high surgical risk group, with an associated high death rate, which is even higher in those with significant co-morbidities.

Peri-operative management: the surgical approach for CHD patients requires a highly skilled multidisciplinary team with broad experience, as anaesthesia can trigger carcinoid crisis and subsequent death in patients going for surgery.^26^,^40^-^57^ The most crucial pre-operative anaesthetic management should encompass optimum control of carcinoid symptoms, and intensified and close monitoring of intra-operative blockade of serotonin receptors. Drugs that may stimulate the release of vasoactive substances from tumour cells should be avoided.^26^,^40^-^57^ The most important drugs to be avoided during the peri-operative period include histamine-releasing neuromuscular relaxants and opioids, as they are associated with detrimental outcomes in CHD patients. The introduction of somatostatin analogues remains a key component to prevent peri-operative carcinoid crisis, and the administration of larger doses of somatostatin analogue is highly recommended in CHD patients.

**Prognosis of CHD**

Previous studies have reported outcome differences between carcinoid syndrome patients with versus those without CHD, and demonstrated markedly shortened life expectancy in patients with underlying cardiac involvement.^8^,^9^ Patients with right heart failure and those with advanced signs of right-sided heart failure represent a subgroup of patients at high risk. Progression of CHD contributes to poor survival; as a result, early detection and regular follow up are mandatory in the management algorithm of patients with CHD.^8^,^9^

Levels of 5-HIAA should be closely monitored, as this is an independent predictor for the development or progression of CHD while on or even after medical management. Although, it was believed that optimal control of serotonin release may prevent or delay development of early cardiac lesions, reports have indicated that somatostatin analogues, chemotherapy and hepatic de-arterialisation may not sufficiently counteract the pathophysiological mechanisms involved in the causation and progression of valvular heart lesions.^8^,^9^

Surgical valve replacement reduces heart-failure symptoms and is associated with improvement in a patient’s functional state. However, a significantly high 30-day mortality rate, which is attributed to bleeding complications, intractable heart failure and other concomitant co-morbidities, has been a major drawback. According to the Duke carcinoid database, supported by other databases, older age, particularly over 60 years of age, is an important independent risk factor for high peri-operative complications and is also associated with high mortality rates.^55^-^59^

## Conclusion

At least half of the patients with carcinoid syndrome present with structural heart disease, usually evidenced during routine transthoracic echocardiographic examination. Although carcinoid heart disease is undoubtedly regarded as a rare form of structural heart disease, a strong suspicion should be raised in patients with classical or florid carcinoid syndrome. Although echocardiography remains the gold standard for diagnostic and confirmatory purposes, biochemical screening and imaging for primary carcinoid tumour should form an integral part of the diagnostic algorithm.

Valve replacement is associated with significant improvement in heart-failure symptoms and improves patients’ functional capacity. However, stringent peri-operative management is vital to avoid carcinoid crisis and impending death. Although there are no definite stringent criteria for the choice of an artificial valve, mechanical valve prostheses are considered durable as they are not affected by vasoactive substances. However bioprosthetic valves should be preferred to avoid long-term anticoagulation. Early detection and valve surgery should be considered to avoid the development of right heart failure, as advanced heart failure represents a high-risk subgroup. In patients with advanced heart failure and possible advanced myocardial dysfunction, transplantation could be an option.
